# Implementation of support vector machines to classify abnormal neuronal response during emotion regulation at an individual level in patients with newly diagnosed bipolar disorder – and its association with subsequent functional changes and mood episodes

**DOI:** 10.1017/S0033291725101876

**Published:** 2025-11-11

**Authors:** Robert James Richard Blair, Alexander Tobias Ysbæk-Nielsen, Hanne Lie Kjærstad, Sahil Bajaj, Klara Coello, Maura Faurholt-Jepsen, Maj Vinberg, Lars Vedel Kessing, Julian Macoveanu, Kamilla Miskowiak

**Affiliations:** 1 Child and Adolescent Mental Health Center, Copenhagen University Hospital – Mental Health Services CPH, Copenhagen, Denmark; 2 Department of Clinical Medicine, Faculty of Health and Medical Sciences, University of Copenhagen, Copenhagen, Denmark; 3 Department of Psychology, Faculty of Social Sciences, University of Copenhagen, Copenhagen, Denmark; 4 Mental Health Services, Neurocognition and Emotion Across Disorders of the Brain Centre (NEAD), Capital Region of Denmark, Denmark; 5 Psychiatric Centre Copenhagen, Copenhagen Affective Disorder Research Centre (CADIC), Copenhagen University Hospital, Frederiksberg, Denmark; 6 Department of Cancer Systems Imaging, Division of Diagnostic Imaging, University of Texas MD Anderson Cancer Center, Houston, TX, USA; 7 The Early Multimodular Prevention and Intervention Research Institution (EMPIRI), Mental Health Centre, Northern Zealand, Copenhagen University Hospital – Mental Health Services CPH, Copenhagen, Denmark; 8 Department of Psychiatry, Virginia Commonwealth University, Richmond, Virginia, USA

**Keywords:** bipolar disorder, emotion regulation, support vector machine, machine learning

## Abstract

**Background:**

In this study, a classifier (hyperplane) is determined to distinguish the neural responses during emotion regulation versus viewing images in healthy adults and then applied to determine (i) the effectiveness of the emotion regulation response (defined as emotion regulation distance from the hyperplane [DFH_ER_]) in independent samples of healthy adults, patients with BD, and the patients’ unaffected relatives (URs) and (ii) the association of DFH_ER_ with the duration of future (hypo)manic and depressive episodes for patients with BD over a 16-month follow-up period.

**Methods:**

Study participants (*N* = 226) included 65 healthy adults (35 used for support vector machine [SVM] learning [HC_Train_] and 30 kept as an independent test sample [HC_Test_]), 87 patients with newly diagnosed BD (67% BD type 2) and 74 URs. BOLD response data came from an emotion regulation task. Clinical symptoms were assessed at baseline fMRI and after 16 months of specialized treatment.

**Results:**

The SVM ML analysis identified a hyperplane with 75.7% accuracy. Patients with BD showed reduced DFH_ER_ relative to the HC_Test_ and UR groups. Reduced DFH_ER_ was associated with reduced improvement in psychosocial functioning during the 16-month follow-up time (*B* = −1.663, *p* = 0.02).

**Conclusions:**

The neural response during emotion regulation can be relatively well distinguished in healthy adults via ML. Patients with newly diagnosed BD show significant disruption in the recruitment of this emotion regulation response. Disrupted may indicate a reduced capacity for functional improvement during specialized treatment in a mood disorder clinic.

## Introduction

Bipolar disorder (BD) is a debilitating psychiatric illness characterized by substantial fluctuations in mood and inter-episode remission (World Health Organization, 1992). Although effective treatments exist, individuals with BD frequently experience residual mood symptoms, impaired functioning (Léda-Rêgo, Bezerra-Filho, & Miranda-Scippa, 2020; Sletved, Ziersen, Andersen, Vinberg, & Kessing, 2023), and lower quality of life (Pascual-Sánchez, Jenaro, & Montes-Rodríguez, 2019). Misdiagnosis is common, and the diagnostic delay between onset and diagnosis is 5–10 years (Baldessarini, Tondo, Baethge, Lepri, & Bratti, 2007; Scott et al., 2022). Moreover, the diagnostic manuals, the Diagnostic and Statistical Manual of Mental Disorders (DSM) and the International Classification of Diseases (ICD), have been challenged with respect to their validity/reliability, the process by which the categories are derived, and the lack of neurobiological underpinnings to these diagnoses (Cuthbert & Insel, 2013; Insel, 2014). This has fueled demands for the development of neurobiology-based biomarkers relevant to psychiatric disorders (the Research Domain Criteria [RDoC] approach; Cuthbert & Insel, 2013, Insel, 2014). Several studies have used machine learning (ML), with various degrees of success, in attempts to determine biomarkers that distinguish patient populations from comparison populations (Dinga et al., 2019; Drysdale et al., 2017; Yang et al., 2018). However, these studies have typically used psychiatric categories as the target for the biomarker. Yet, there are concerns with the reliability of many psychiatric diagnoses (Regier et al., 2013). As such, the RDoC approach suggests an alternative strategy – a focus on dimensions of neuro-cognitive function and a determination of the extent to which perturbations of such function are associated with groups of psychiatric symptoms.

One neuro-cognitive function of clear relevance to BD is emotion regulation (for a review, see Kurtz, Mohring, Förster, Bauer, & Kanske, 2021, Miskowiak et al., 2019). Emotion regulation involves dampening, amplifying, or maintaining emotions both before or after they arise (Gross, 1998). At the neural level, effective emotion regulation relies on the interaction between prefrontal cognitive control regions and emotion-generating limbic regions (Buhle et al., 2014; Morawetz, Bode, Baudewig, & Heekeren, 2017). Studies in patients with BD have consistently shown aberrant neural activation and connectivity during explicit down-regulation of negative emotion (for reviews of this literature, see Ahmed et al., 2023, Kurtz et al., 2021, Miskowiak et al., 2019, Townsend & Altshuler, 2012). Specifically, patients with BD exhibit heightened amygdala activation (Corbalán, Beaulieu, & Armony, 2015; Kanske, Schönfelder, Forneck, & Wessa, 2015) and hypo-activity within the dorsolateral and the ventrolateral prefrontal cortex (DLPFC, VLPFC) (e.g. Kjærstad et al., 2023). There have also been reports that adult unaffected relatives (URs) of patients with BD also demonstrate reduced activity in these prefrontal regions during emotional regulation (Kanske et al., 2015; Kjærstad, Eikeseth, Vinberg, Kessing, & Miskowiak, 2021; Meluken et al., 2019). Furthermore, emotion dysregulation may have prognostic implications, with maladaptive strategies (e.g. rumination, self-blame) being associated with higher levels of depressive symptoms (Fletcher, Parker, & Manicavasagar, 2014) and depressive episodes (Alloy et al., 2009), while adaptive strategies (e.g. cognitive reappraisal) are associated with lower levels of depression (Johnson, Tharp, Peckham, & McMaster, 2016). Notably, hypo-activity in the dorsomedial prefrontal cortex during emotion regulation has been associated with an increased likelihood of subsequent episode relapse during a 16-month follow-up (Kjærstad et al., 2022a).

Before a dimensional marker of a neuro-cognitive function can have clinical utility, it must be possible to calibrate the function’s effectiveness at the level of the individual participant (i.e. the degree to which the individual’s neural response corresponds to the neural signal associated with that neuro-cognitive function). Standard univariate analysis approaches have proven highly effective in identifying functional abnormalities in patient groups and providing information on the localization of function (e.g. Blair et al., 2022a; Buhle et al., 2014; Corbalán et al., 2015; Kanske et al., 2015; Kjærstad et al., 2023; Morawetz et al., 2017). However, they risk what has been termed ‘blobology’; i.e. a lack of clarity with respect to determining the extent to which a regional activation in one study is replicated in another (e.g. Hanson, 2022). Moreover, it is difficult to determine unitary indices of function effectiveness for individual participants from univariate analyses. Recently, there have been suggestions that multivariate analyses might provide greater hope with respect to the calibration of an individual’s function effectiveness (Blair et al., 2024; Blair, Mathur, Haines, & Bajaj, 2022b; Han et al., 2022). Based on a healthy training sample, support vector machine (SVM) learning might be used to classify neurocognitive functions (e.g. emotion regulation versus passive negative picture viewing). Such a classifier could then be used to determine an individual’s distance from the hyperplane (DFH) during emotion regulation (see Supplemental Material 1 and Supplemental Figure 1). DFH, by indicating the degree to which the SVM considers the data point to belong to a particular class of neuro-cognitive function, can be considered to index functional effectiveness (i.e. the degree to which the individual’s neural response corresponds to the neural signal that the model is confident corresponds to that neuro-cognitive function).

In this study, we determine an SVM derived classifier for distinguishing the neural response during emotional regulation relative to passively viewing negative images. Our first goal was to use this classifier to determine the extent to which patients with BD show a significantly weak emotional regulation response (i.e. a reduced distance of their emotional regulation response from the hyperplane; for greater details of our theoretical view, see Supplemental Material 1). Our second goal was to determine the extent to which emotion regulation distance from hyperplane (DFH_ER_) was associated with changes in psychosocial functioning and mood episode relapse in patients with BD over a 16-month period. Based on the previous literature, we predicted that (i) brain responses to emotion regulation versus passive viewing of negative images would be accurately classified in healthy adults; (ii) the identified pattern of neural recruitment distinguishing these functions would implicate lateral and dorsomedial frontal cortex (emotion regulation) and the amygdala (view negative); (iii) patients with BD and perhaps also URs would show reduced distance from the hyperplane for emotion regulation trials relative to healthy comparison adults; and (iv) low function effectiveness during emotion regulation trials would be associated with less ability to show improvements in psychosocial functioning in response to optimized treatment and more relapse episodes within the subsequent 16-month follow-up time.

## Method

### Study design and participants

The study is based on the cohort investigated by Kjærstad et al. (2021, 2022a), including baseline data from patients with newly diagnosed BD and their unaffected first-degree relatives (UR) and healthy controls (HCs) recruited as part of the Bipolar Illness Onset (BIO) study (Kessing et al., 2017). Study participants included 87 patients with BD, 74 UR of these patients, and 65 HC. The HCs were divided into two samples, 35 used for the SVM ML (HC_Train_) and 30, independent of the first group as a test sample (HC_Test_). Participants underwent an fMRI investigation at baseline with clinical data and ratings of psychosocial functioning collected in conjunction with the baseline fMRI scan and 16 months later. Participants’ global functioning was assessed with the Functional Assessment Short Test (FAST; Rosa et al., 2007). For further details on the clinical assessment, recruitment, and inclusion criteria, see Supplemental Material 2.

## Measures

### Emotion regulation paradigm

The fMRI task employed in this study was a well-established voluntary emotion regulation paradigm (Banks, Eddy, Angstadt, Nathan, & Phan, 2007), involving the presentation of neutral and negative images from the International Affective Picture System (IAPS; Lang, Bradley, & Cuthbert, 1997) that has been described in our previous work (Kjærstad et al., 2021; Kjærstad et al., 2023; Kjærstad, Poulsen, Vinberg, Kessing, & Miskowiak, 2022b). Participants were instructed to view the images in the ‘view’ conditions and to dampen their emotions in the ‘dampen negative’ conditions. See Supplementary Material 3 for further details.

### Pre-processing of fMRI data and region of interest determination

The fMRI data were processed using FEAT (FSL version 6.0.5.2). Functional and structural volumes underwent visual quality assessment to exclude low-quality datasets. The preprocessing pipeline included brain extraction, rigid-body motion correction, linear registration to the individual’s T1-weighted image, non-linear registration to standard MNI space at 2 mm isotropic voxel size, and spatial smoothing with a 5 mm FWHM Gaussian kernel.

Subject-level analysis employed a general linear model (GLM) with three explanatory variables (EVs): ‘view neutral’, ‘view negative’, and ‘dampen negative’. EVs were convolved with a double-gamma hemodynamic response function, and temporal derivatives were modeled to correct for slice-timing effects. Six motion parameters were included to account for head movement. Movement outliers exceeding 0.20 mm mean relative displacement, determined by MCFLIRT, were visually inspected to ensure image quality.

A functionally derived 400-parcellation cortical atlas aligned with the MNI152 2 mm template was utilized to define 400 regions of interest (ROIs) using the fslmaths function in FSL. Additionally, five subcortical regions from the Harvard-Oxford probabilistic subcortical atlas were included based on a priori hypotheses: the left and right caudate, amygdala, hippocampus, nucleus accumbens, and putamen.

For further details on statistical analyses of demographic and clinical data, pre-processing and first-level analysis of fMRI data, preparation of the regions of interest and Signal change extraction and missing data protocol, see Supplemental Material 3.

### Feature selection and machine learning analysis

The fMRI data from 35 HC was used to determine the features selected to make up the hyperplane differentiating the BOLD response to the emotion regulation and view negative trials. We took the K_1_ 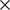 K_2_ nested cross-validation approach to estimate model performance (Varma & Simon, 2006), where K_1_ and K_2_ represent the number of outer and inner loops respectively. We chose K_1_ = 10 and K_2_ = 10 in the current study. There is no formal rule regarding the values for K_1_ or K_2_, however, both the 10-fold and the 5-fold cross-validations have been shown empirically to yield test error estimates that maintain a balance between excessively high bias and excessively high variance (Kuhn & Johnson, 2018). The 10-fold cross-validation is more commonly used than the 5-fold cross-validation because of better computational efficiency. In the outer loop, the randomized data were split into K_1_ folds where K_1_–1 folds were used as the training data and one-fold was used as the test data. The training data were transformed into z-score, and the corresponding transformations were used to the testing data. The least absolute shrinkage and selection operator (LASSO; Tibshirani, 1996) (function *lasso* from MATLAB R2021a) was used on the training data to select features. This procedure was repeated K_1_ times, each time leading to a set of best features. The final set of features was formed by the features that appeared at least 50% times in the K_1_ sets of best features. The features that were not in this final set were excluded from the training and test data. In the inner loop, the reduced training data were further divided into K_2_ folds, i.e. K_2_–1 folds were used as the sub-training data and one-fold was used as the validation data. The inner loop was repeated K_2_ times. In each iteration, the SVM ML classifier was trained using the sub-training data. The obtained model was then tested on the validation data resulting in a value of validation accuracy. The hyperparameters in the model were tuned using Bayesian optimization during each iteration to maximize validation accuracy for each model. The performance of the model which gave the highest validation accuracy from the inner loop was then evaluated on the test data left in the outer loop. This step was repeated K_1_ times. The average of the K_1_ test-accuracies computed from the outer testing folds was regarded as the *generalized accuracy.* The corresponding sensitivity and specificity parameters were regarded as *generalized sensitivity* and *generalized specificity* respectively. All the steps for feature selection and ML analysis were performed in MATLAB R2022b (for a depiction of this ML framework, see Figure 2; Bajaj et al., 2023).

Following this, *distance from the hyperplane* (DFH) was calculated for each participant for both their response during emotion regulation and during viewing negative images. The DFH was calculated for all participants (HC_Train_, HC_Test_, UR, and patients with BD).

**Demographic, clinical, and behavioral (emotion regulation rating) data:** Potential group differences in these data were assessed via one-way ANOVAs or chi-squares.

**Group differences in DFH as a function of task (dampen versus view negative):** A 3 (Group: HC_Test_, BD, UR) × 2 (Task: Emotion regulation, View negative) ANOVA was conducted on the DFH data.

**Sensitivity analyses for group differences in DFH as a function of task:** Given the potential for group differences in demographic variables, sensitivity analyses were planned; the 3 × 2 ANOVA was repeated as an ANCOVA with covariates being demographic variables significantly differing the groups. In addition, the ANOVA was repeated excluding patients with BD receiving medication.

**Prognosis as a function of DFH:** To determine whether emotion regulation DFH was associated with total duration of subsequent mood episodes and decreases in functioning (baseline to follow-up), we employed two stepwise multiple regression analyses with total number of relapse episodes (depression and mania) and change in functioning (subtracted total FAST follow-up score from baseline score) as the outcome variables. DFH for emotion regulation and view negative trials were entered as variables. Analyses were conducted within the entire BD sample, using data available at both time points for relapse episodes (*N* = 58) and FAST data (*N* = 50), and controlling for baseline severity of affective symptoms (measured via the Hamilton Depression Rating Scale [HDRS]) and the Young Mania Rating Scale [YMRS]), age, sex, BD type (BD type 1 or BD type 2), and duration between the scan and follow-up. All analyses were conducted using SPSS version 25.

## Results

### Missing data and exclusions

Out of 228 participants, three were excluded due to excessive movement.

### Demographic, clinical, and behavioral data

Baseline demographic and clinical characteristics are presented in Table 1. The 87 patients with BD (67% BD type II), their 73 URs, the 30 HC_test,_ and the 35 HC_train_ were comparable with regards to sex and years of education (*p*-values ≥ .13). There were statistically significant differences between the four groups for (i) age (*p* = .04), which was driven by BD patients being older than UR and HC_test_ (*p*-values ≤ .049) and (ii) subsyndromal depression and mania symptoms and levels of functioning (*p*-values < .001): patients with BD presented with more baseline depression and mania symptoms and poorer functioning compared to UR, HC_test,_ and HC_train_ (*p*-values < .001). No differences in clinical characteristics or functioning were observed between BD types I and II (*p*-values>.28). URs also had poorer functioning compared to both HC groups (*p*-values ≤ .02). There were no significant differences between the HC_test_ and HC_train_ samples.

**Table 1.** Baseline demographic and clinical characteristics in patients with recent diagnosed bipolar disorder (BD), their unaffected relatives (UR) and healthy controls test (HC test) and healty Control train (HCtrain)

	BD	UR	HC_test_	HC_train_	F/χ^2^	P-value	Pairwise comparisons
*n*	87	73	30	35			
Sex, *n* (% female)	60 (69%)	37 (51%)	19 (63%)	22 (61%)	5.66	0.06	
Age	29.8 [7.42]	27.5 [7.09]	27.2 [7.78]	31.9 [10.46]	2.51	0.08	
Years of education	15.1 [4.12]	15.0 [2.83]	15.9 [1.91]	15.7 [2.52]	0.66	0.52	
HDRS	5.3 [4.14]	1.6 [2.40]	1.0 [1.19]	1.0 [1.18]	34.19	**<.001**	BD > UR & HC_test_ & HC_train_
YMRS	2.7 [3.08]	0.8 [1.53]	0.8 [1.94]	0.7 [1.56]	13.45	**<.001**	BD > UR & HC_test_ & HC_train_
FAST, total score	15.6 [11.33]	4.0 [6.98]	1.1 [1.85]	1.2 [1.53]	47.83	**<.001**	BD > UR > HC_test_ & HC_train_
Bipolar type, *n* (% type 2)	58 (67%)						
Illness duration, years	8.5 [7.81]						
Antidepressants, *n* (% yes)	19 (22%)						
Antipsychotics, *n* (% yes)	18 (21%)						
Anticonvulsants, *n* (% yes)	32 (37%)						
Lithium, *n* (% yes)	41 (47%)						
Dampening (mean, s.d.)	0.39 (0.71)	0.41 (0.63)	0.63 0.75)	0.67 (0.61)	F = 3.36	0.04	HC_all_ > BD & UR

Notes: Group differences assessed with Kruskal–Wallis *H* test and chi-square analyses. Values are reported as standard deviation [] and *n* (%).HDRS = Hamilton Depression Rating Scale; YMRS = Young Mania Rating Scale; FAST = Functioning Assessment Short Test; Dampening = The extent to which the patient’s reduced their rating of the unpleasantness of the images in the dampen condition relative to the view negative condition.

*Emotional dampening:* With respect to the task, a one-way ANOVA revealed significant differences in emotion dampening (*F*(2, 218) = 3.35, *p* = 0.037, 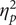 = .03); see Table 1. The HC participants showed greater regulation than the BD patients and URs (*t*(148) & *t*(134) = 2.34 & 2.19, *p* = 0.02 & 0.03, 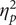 = .036 & 0.035 respectively) who did not significantly differ (*t*(154) = 0.19, *p* = 0.85 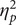 = .000); see Table 1.

### Generalized model performance and feature identification

With respect to the BOLD response data for response to dampening emotions versus viewing negative images, our SVM ML analysis identified a hyperplane during training with accuracy = 75.7%, sensitivity = 74.8%, and specificity = 74.7%. Accuracy of the hyperplane for dampen and view negative for the three independent samples (HC_test_, URs, and BD patients) was comparable (75.0%, 68.9%, and 64.5%, respectively); χ^2^(df[2] = 2.35, *p* = 0.31. However, there were group differences in sensitivity for emotion regulation, though not specificity, for ‘view negative’ conditions (see Supplemental Table 1).

**Figure 1.**
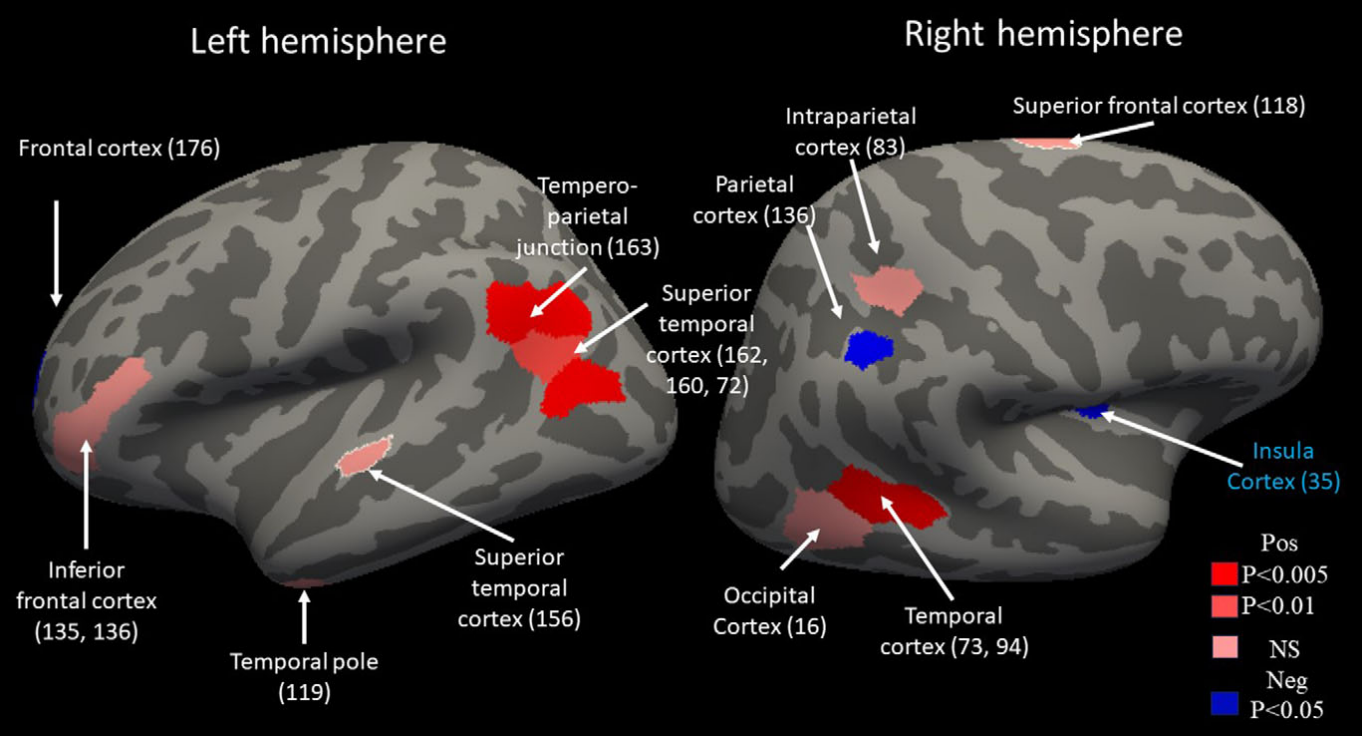
The features selected from the healthy training sample. The features, identified via LASSO (see Supplemental Materials) differentiating the BOLD response as a function of dampen negative versus view negative. Regions in red showed a positive [Pos] association between activity and DFH_Dampen_. Regions in blue showed a negative [Neg] association between activity and DFH_Dampen_. Numbers correspond to Schaefer region numbers.

A total of 18 features were identified (see Figure 1 and Supplemental Table 2), 14 of these features (including dorsomedial and inferior frontal cortices) showed greater responses to dampen relative to view negative while four features (including medial temporal and intraparietal cortices) showed significantly greater responses to view negative relative to the dampen condition (see Figure 1).

### Group differences in DFH as a function of task (dampen versus view negative)

**Figure 2.**
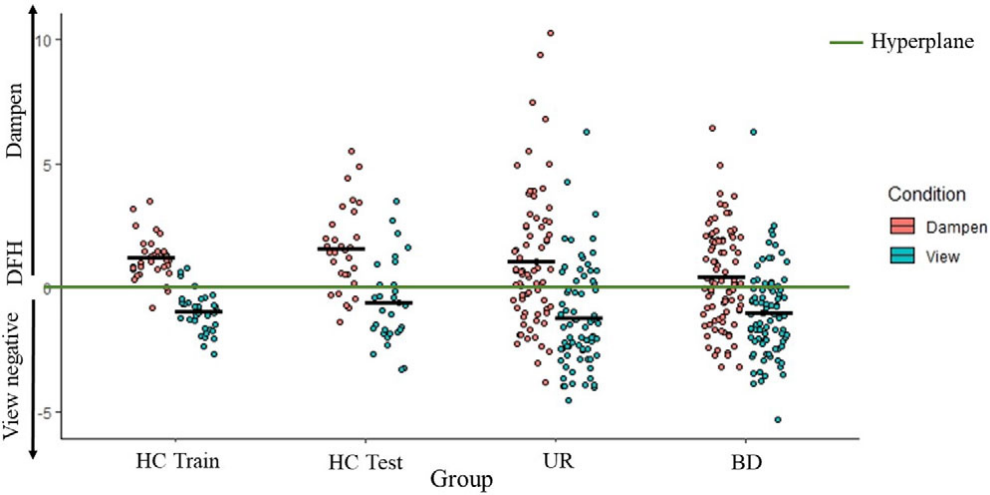
Distance from hyperplane (DFH) for dampen and view negative as a function of group. Groups are healthy comparison training sample [HC_Train_], healthy comparison test sample [HC_Test_], the patient’s unaffected relatives (UR), and the patients with bipolar disorder (BD). Dampen refers to the extent to which the patient’s reduced their rating of the unpleasantness of the images in the dampen condition relative to the view negative condition.

The three (Group: HC_Test_, BD, UR) × two (Task: Emotion regulation, View negative) ANOVA on the DFH data revealed a significant Group-by-Task interaction (*F*(2, 187) = 7.17, *p* = 0.001, 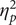 = .071); see Figure 2. Follow up 2 (Group) × 2 (Task) ANOVAs revealed significant interactions for both HC_Test_ vs BD and UR vs BD (*F*(1, 114) & *F*(1, 158) = 5.23 & 12.11, *p* = 0.024 & 0.001, 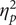 = .044 & 0.071). However, there was no significant interaction for HC_Test_ vs UR (*F*(1, 102) = 0.14, *p* = 0.707, 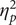 = .001); see Figure 2. See Supplemental Material 4 for additional analyses including HC_Train_.

### Sensitivity analyses for group differences in DFH as a function of task

Given group differences in age, depression, and mania symptoms, the three (Group: HC_Test_, BD, UR) × 2 (Task: Emotion regulation, View negative) ANOVA on the DFH data was repeated as an ANCOVA, including age, HDRS, and YMRS scores and FAST total score as covariates. The Group-by-Task interaction remained significant (*F*(2, 179) = 3.68, *p* = 0.027, 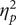 = .04).

Given some of the patients with BD received medications (see Table 1), the three (Group: HC_Test_, BD, UR) × two (Task: Emotion regulation, View negative) ANOVA on the DFH data was re-run in a sensitivity analysis excluding all participants with BD on medication (*N* = 19 unmedicated). The Group-by-Task interaction remained significant (*F*(2, 120) = 3.44, *p* = 0.035, 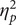 = .054), indicating that the findings were not confounded by effects of medication. Among BD, there were no differences between BD type I and II in DFH during emotion regulation or view negative (*p*-values>.14. For additional medication analyses, see Supplemental Table 4.

### Change in functioning and number of relapse episodes as a function of DFH

**Figure 3.**
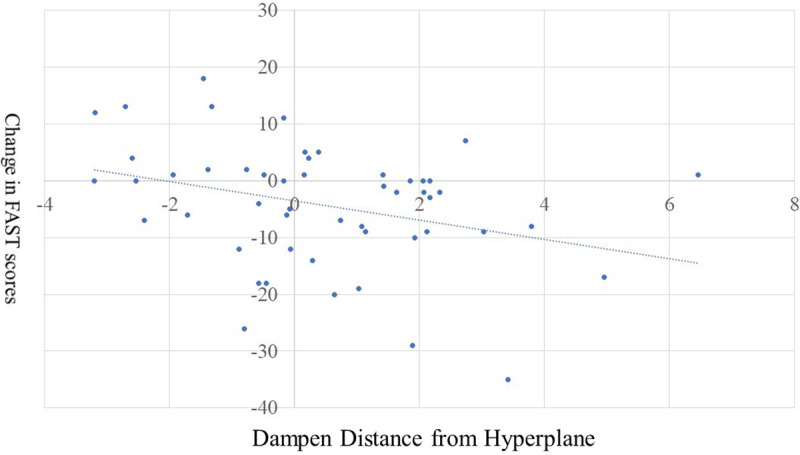
The association of dampen distance from hyperplane and change in FAST scores. Dampen refers to the extent to which the patient’s reduced their rating of the unpleasantness of the images in the dampen condition relative to the view negative condition.

Within the patients with BD, the stepwise regression analysis for change in functioning was significant (*R* ^2^ = .11, *F*(1,48) = 5.61, *p* < .02). DFH_ER_ was associated with change in functioning (*B* = −1.663, *p* = 0.022); increased emotion regulation DFH was associated with more improvement in functioning (see Figure 3). Note that removal of the patient who was an outlier in their response during emotion regulation, increased the significance of this finding (*B* = −1.865, *p* = 0.006). In contrast, DFH for emotional regulation trials was associated with total number of relapse episodes (for additional details of these analyses, see Supplemental Table 5).

## Discussion

In this study, we used SVM to develop an emotion regulation classifier (dampen relative to viewing negative images) from healthy adult BOLD response data (HC_Train_). Our goals were to determine the extent to which (i) patients with BD versus URs and healthy adults (HC_Test_) might show differences in DFH for emotion regulation and view negative data and (ii) DFH for emotion regulation and/or view negative data might be associated with prognosis, in terms of time spent in mood episodes and changes in functioning, in patients with BD. There were three main findings: First, a hyperplane distinguishing the BOLD response to emotion regulation versus view negative trials could be identified that was accurate in identifying the emotional regulation and viewing negative activations in an independent sample of healthy adults as well as URs of patients. Second, there were significant group differences in DFH for emotion regulation and view negative data – specifically for patients with BD (independent of subtype) vs both URs and healthy adults. Third, there were significant positive associations between emotion regulation DFH (DFH_ER_) and the change in global functioning from baseline to follow-up (though not the number of relapse episodes) even after controlling for baseline demographic and symptom severity variables.

The approach developed in the current paper operationalizes an objective ML-based method for identifying neuro-cognitive systems involved in specific task-based functions that may be compromised in psychiatric patients. The classifier developed from the healthy adult data (HC_Train_) was reasonably successful in identifying the emotional regulation and view negative activations in the test samples (HC_Test_, UR, and, to a lesser extent, patients with BD). Notably, the features making up the hyperplane mirrored those previously identified in the literature. Features identified included dorsomedial and inferior frontal cortices (see Figure 1). These are regions implicated emotion regulation via the meta-analytic review by Buhle and colleagues (2014). It should be noted that one region that was not identified as a feature was the amygdala. The amygdala is frequently seen to show greater responses during view negative relative to emotion regulation trials (Buhle et al., 2014). That it was not identified here may reflect the specifics of this emotion regulation paradigm or the relatively small N of the training sample (see also below).

Our first goal was to determine the extent to which patients with BD vs URs and healthy adults (HC_Test_) might show differences in DFH for emotion regulation and view negative data. Notably, there was a highly significant group × task interaction that follow-up analysis indicated was insensitive to potential confounds. Patients with BD showed less DFH differentiation between emotion regulation and viewing negative trials than both URs and HC_Test_. These results were primarily driven by reduced DFH_ER_ in the patients with BD. Previous work has indicated deficient neural recruitment during emotion regulation in patients with BD (e.g. Gould & Gottesman, 2006; Kjærstad et al., 2021; Kurtz et al., 2021; Miskowiak et al., 2019). The current data are consistent with this.

Our second goal was to determine the extent to which the DFH_ER_ (i.e. emotion regulation function effectiveness) might be associated with relapse and improvements in global functioning in patients with BD. The current results show that the DFH of the emotion regulation response at baseline was associated with greater improvement in global functioning but not with a reduced number of relapse episodes from baseline to follow-up. Previous work using seven ROIs within the frontal cortex derived from healthy participant’s engaged in emotional regulation found that hypo-activity during emotion regulation in one of these regions (left dmPFC) was associated with a greater likelihood of subsequent relapse (Kjærstad et al., 2022a). While we found no such association with future relapse, our observations indicate that the effectiveness of the neuro-cognitive emotion regulation system might serve as a viable treatment target in BD to improve functioning. At the time of the assessment, patients were recently diagnosed and were enrolled in specialized care at the Copenhagen Affective Disorder Clinic, which may explain their overall functional improvement over the 16-month follow-up time (on average) observed at a group level (Kjærstad et al., 2022a). It was therefore a potentially clinically important observation that less functional integrated response during emotion regulation was associated with *failure to* improve functionally over this follow-up time. This could therefore serve as a neurocircuitry biomarker at an individual level for patients’ likelihood of achieving functional rehabilitation and be useful for selection of participants into trials that specifically focus on targeting functional impairments in BD, such as functional remediation programs (Torrent et al., 2013; Valls et al., 2021).

While this study has several strengths including the well-defined newly diagnosed patients with BD and their unaffected relatives, the use of a well-established fMRI task assessing emotion regulation and a novel approach to analysis, several limitations should be considered. First, our HP_train_ sample was relatively small (*N* = 35). Increasing the size of the training dataset has advantages in machine learning analyses (e.g. Koppe, Meyer-Lindenberg, & Durstewitz, 2021). However, it is important to note that training set *N* is partly a function of data complexity (Koppe et al., 2021) and the critical question is applicability of the obtained hyperplane to independent datasets. Importantly, the hyperplane determined from the HP_train_ allowed the characterization of the data of the HP_test_. Second, recent work has seriously challenged the use of contrast-based data, rather than individual regressor based analyses, in individual differences research because of data reliability concerns. It is important to note here though that group-based analyses are relatively insensitive to the reliability concerns of contrast-based analyses (see Blair, Mathur, et al., 2022b; Chen et al., 2021). Moreover, the determination of the hyperplane was derived from a group-based analysis. The follow-up analyses were individual difference-based analyses but importantly they were based on a single condition; distance of the reward response from the hyperplane – not a contrast against another condition (i.e. DFH_ER_-DFH_ViewNeg_). Third, while the patients were ‘newly diagnosed’ at baseline, they had been ill for a median of 5 years (quartiles: 3–13) prior to receiving treatment for BD (Table 1). Thus, we cannot exclude the neuro-progressive effects of prior mood episodes and years of untreated illness, the association of atypical emotion regulation function and change in FAST scores during follow-up, may reflect the association of both with severity of prior illness. Fourth, it could be argued that the reduced emotion regulation DFH of the patients with BD represented a lack of motivation to engage with the task rather than a difficulty in neural recruitment. Certainly, the HC participants behaviorally reported greater dampening of their emotional responses than the patients with BD. However, it is notable that the levels of dampening of the patients with BD and their URs did not significantly differ. Yet, the patients with BD showed significantly reduced DFH_ER_ than their URs; i.e. despite similar levels of self-reported emotion regulation, the patients with BD were still showing difficulties, relative to their URs, in their recruitment of an emotion regulation neural response (though not in their recruitment of a response to viewing negative images). Fifth, it could be argued that it would be better to train a model on a mixture of bipolar, unaffected relatives, and controls. In the interests of space, we consider this issue further in Supplmental Material 5. Sixth, it is possible that an increase in the number of features might increase the accuracy of the model. However, increasing the number of features with our relatively small training n might be unwise due to the danger of over-fitting. However, future work might involve a larger training n. Sixth, the use of imputation could have led to data leakage (see Rosenblatt, Tejavibulya, Jiang, Noble, & Scheinost, 2024). However, it is important to note that this impacted only 21 subjects and 7 ROIs (45 total cells across 21 subjects, i.e. 0.00012% of the data).

In conclusion, we identified a physiological range for the pattern of brain responses to emotion regulation versus passive view of negative images, which could be validated in an independent sample of healthy adults as well as URs of patients. Patients with BD differed significantly from HCs on their estimated function effectiveness for emotional regulation, with significantly more patients showing abnormal pattersns of neural responses. Importantly, patients’ function effectiveness (distance from hyperplane) was associated with the lack of functional rehabilitation during their specialized care in the 16-month follow-up time, even after controlling for baseline demographic and symptom severity variables. The distance from the hyperplane of neural response during emotion regulation measure could, therefore, be an important biomarker at an individual level for treatment stratification and consideration of a patient’s prognosis for functional recovery.

## Supporting information

Blair et al. supplementary material Blair et al. supplementary material

## Supplementary material

The supplementary material for this article can be found at http://doi.org/10.1017/S0033291725101876.
