# Supplementary material for: Implementation of support vector machines to classify abnormal neuronal response during emotion regulation at an individual level in patients with newly diagnosed bipolar disorder – and its association with subsequent functional changes and mood episodes
Source: Psychol Med. 2025 Nov 11;55:e338. doi: 10.1017/S0033291725101876 (PMC13058661; doi:10.1017/S0033291725101876)
Supplement: Blair et al. supplementary material [file S0033291725101876sup001.docx]

**Supplemental Material**

1. **Further details on the theoretical underpinnings of the approach**

*Developing the hyperplane from HC_Train_:* Considerable data indicates that the neural response during emotion regulation can be distinguished from the neural response during viewing negative images (for a review of the literature, see Buhle et al., 2014). As such, it can be hypothesized that a Support Vector Machine (SVM) can be used to identify a hyperplane distinguishing these responses in healthy participants.

The SVM aims to find the hyperplane that best separates two classes (here neural responses to emotion regulation vs. responses during viewing negative images); i.e., the hyperplane that maximizes the distances from the support vectors corresponding to individual participants’ responses during emotion regulation and when viewing negative images (see Supplemental Figure 1). In Supplemental Figure 1, there are only two features examined (X_1_ and X_2_). However, the SVM conducted on the current data is multidimensional (and led to a hyperplane involving 18 features).

*Distance from hyperplane (DFH):* We consider that DFH, by indicating the degree to which the SVM considers the data point to belong to a particular class of neuro-cognitive function, can be considered to index function effectiveness (i.e., the degree to which the individual’s neural response corresponds to the neural signal that the model is confident corresponds to that neuro-cognitive function).

We conceptualize our approach similarly to the neuropsychological approach. Neuropsychological tasks are designed to test the efficiency of putative neurocognitive functions. Tasks are developed to measure the neurocognitive function, they are tested with healthy participants and then examined to determine: (i) if performance replicates in an independent group of healthy participants; and (ii) the extent to which patients with neurological damage in the putative substrate show significant impairment on the task (or are outside the healthy normative range). One could argue that our SVM approach is overfitted for HC participants and it is true that our approach, like the neuropsychological approach, begins with HC data but we would contend that the field does not typically consider neuropsychological tasks overfitted for HC participants.

*A consideration of misclassifications:* The current tasks contrasts dampening emotional responding with emotional responding. An individual failing to dampen their emotional response should engender a relatively larger emotional response. As such, we would argue, in this task, misclassifications of emotion regulation are being “correctly classified” as the opposite indexed response (emotional signaling) and -ve values for DHF_Dampen_ reflect emotional responding (i.e., the difficulty faced by the patient). This reasoning would not follow though if we were classifying functions that should not be considered antagonistic to each other (e.g., reading and maths) - it would be difficult to interpret opposite direction DFHs for these functions.

*A note regarding the possibility that patients with BD use different neural systems that healthy adults during dampening emotional responding or viewing negative images:* The extent to which patients with BD use different neural systems during dampening emotional responding or viewing negative images is an interesting theoretical question, albeit one not addressed in the current paper. Our data address questions regarding the extent to which patients use the neurocognitive system that healthy participants use for these functions and the extent to which failure to use these circuits is associated with clinical outcome. As such, our research question is orthogonal to the question regarding whether patients with BD use a different circuit to achieve a poorer level of emotion regulation.

Our research questions are based within a Research Domain Criteria approach; i.e., they assume that there are neurocognitive systems in healthy participants that perform specific functions and that, when these systems are disrupted, psychiatric symptoms may emerge. This theoretical approach motivates our questions regarding whether we can use SVM to index the efficiency of these systems in HCs, do patient with BD show reduced functional efficiency and is functional efficiency associated with patient’s clinical outcomes.

An alterative strategy is the more classic group-based approach, i.e., to what extent can you differentiate patient groups based on their neural signature? Such questions are interesting and ones that we have addressed in other papers, but they are not the questions we were interested in addressing in this paper.

It will be extremely interesting to see the extent to which the two approaches achieve clinically useful data/indices. We believe that our RDoC orientated neuropsychological type approach will prove useful. But it is quite possible that the currently dominant group-differentiation indices approach will prove more clinically useful. It will be an empirical question.

1. **Further details on the clinical assessment, recruitment and inclusion criteria and task**

The clinical assessment consisted of a semi-structured interview based on the Schedules for Clinical Assessment in Neuropsychiatry (SCAN) (Wing et al., 1990) by MDs or MSc in psychology to ascertain diagnosis status upon inclusion in the study. The recruitment of individuals with BD was made exclusively from the Copenhagen Affective Disorder Clinic, where the diagnosis was given within 2 years prior to study enrolment. With regards to showcasing the heterogeneity of the disorder, all patients referred to the clinic between aged 18 and 60 years, after having received a BD diagnosis, were eligible and thus asked to participate in the study. Individuals were diagnosed with BD according to the SCAN interview using International Classification of Diseases (ICD-10) criteria (WHO, 1992). Patients’ URs were recruited after patient consent. The URs comprised of siblings and offspring of patients, aged 15-40 years, who had no personal lifetime history of mental disorders or substance use disorder ascertained with the SCAN.

For the healthy control group, age- and sex-matched individuals were recruited from the University Hospital, Rigshospitalet, Blood Bank. Exclusion criteria were personal or family (first-degree relatives) history of mental disorders or substance abuse. Lack of first-degree familial history of psychiatric illness was ascertained by thorough questioning into participants' familial history.

For all participants inclusion criteria included total score ≤ 14 on both the Hamilton Depression Rating Scale (HDRS-17) (Hamilton, 1967) and the Young Mania Rating Scale (YMRS) (Young, Biggs, Ziegler, & Meyer, 1978), and exclusion criteria were a history of severe brain injury, neurological disorder (including dementia), current severe somatic illness, and/or substance abuse disorder. Participants’ level of functioning was assessed with the Functional Assessment Short Test (FAST; Rosa et al., 2007). Patients’ history of mood episodes was assessed at both baseline and follow-up and type of episode (hypomanic, manic, depression, mixed), number and duration of episodes were registered.

The authors of this paper declare that all procedures contributing to this work comply with the ethical standards of the relevant national and institutional committees on human experimentation and with the Helsinki Declaration of 1975, as revised in 2008. The study was approved by the Committee on Health Research Ethics of the Capital region of Denmark (protocol number: H-7-2014-007) and the Danish Data Protection Agency, Capital Region of Copenhagen (protocol number: RHP-2015-023). Informed consent was obtained for all participants prior to study participation.

**Emotion regulation paradigm:** The fMRI task employed in this study was a well-established voluntary emotion regulation paradigm (Banks, Eddy, Angstadt, Nathan, & Phan, 2007), involving the presentation of neutral and negative images from the International Affective Picture System (IAPS; Lang, Bradley, & Cuthbert, 1997) that has been described in our previous work (Kjærstad, Eikeseth, Vinberg, Kessing, & Miskowiak, 2021, Kjærstad et al., 2023, Kjærstad, Poulsen, Vinberg, Kessing, & Miskowiak, 2022). The task comprised 24 neutral and 48 unpleasant pictures, presented in three conditions: passive view of neutral images (‘passive view neutral’, four images), passive view of unpleasant images (‘passive view negative’, four images), and a voluntary downregulation condition that involved only unpleasant images (‘dampen negative’, four images), resulting in a total task time of 12 min. Participants were instructed to view the images in the ‘view’ conditions and to dampen their emotions in the ‘dampen negative’ conditions. Each of the three conditions was presented randomly six times, interleaved by a 16 s fixation cross on a blank screen. Each condition included different set of pictures and started with an instruction to “view” or “dampen” (4 s), followed by the presentation of four corresponding images (4 s), and concluded with a rating of unpleasantness (4 s) on a range from1 (not at all unpleasant) to 5 (very unpleasant) (4 s), indicated by participants by using a button box with five buttons with their right hand. We used different sets of unpleasant images in the ‘view negative’ and ‘dampen negative’ conditions, which were matched for valence (p = 0.54) and arousal (p = 0.56) according to the IAPS normative ratings (Lang et al., 1997).

1. **Further details on the statistical analyses**

**Statistical** **analyses of demographic and clinical data**

Data normality distribution was explored using Shapiro-Wilk test. To assess differences in baseline demographic and clinical characteristics between BD patients, their URs, HC_test,_ and HC_train,_ we employed non-parametric Kruskal-Wallis H test and Pearson's chi-square (χ^2^).

**Pre-processing and first-level analysis of fMRI data**

The fMRI data was processed using the FEAT (Woolrich, Ripley, Brady, & Smith, 2001) tool of the FMRIB Software Library (FSL), version 6.0.5.2 (Jenkinson, Beckmann, Behrens, Woolrich, & Smith, 2012). Prior to analysis, visual quality assessment of functional and structural volumes was performed to exclude low-quality datasets. The pre-processing pipeline encompassed brain extraction, rigid-body motion correction, linear registration to the individual T1-weighted image, non-linear registration to the standard MNI (Montreal Neurologic Institute) space at 2mm isotropic voxel size, and spatial smoothing using a 5mm full-width-half-maximum gaussian kernel.

A general linear model (GLM) was implemented for subject-level analysis which included three explanatory variables (EVs) to model the task: ‘view neutral’, ‘view negative’, and ‘dampen negative’. The EVs were convolved with a double-gamma hemodynamic response function, and we modeled temporal derivates to correct for slice-timing effects. In addition, six basic subject-level movement regressors were included to account for head movement. Movement outliers exceeding 0.20 mm in mean relative displacement, as calculated by the MCFLIRT (Jenkinson et al., 2012) tool within FSL, were visually inspected to ensure image quality.

**Preparation of the regions of interest**

A functionally derived 400-parcellation cortical atlas fitted to the MNI152 2mm template (Schaefer et al., 2018) was used to separate out 400 regions-of-interest (ROIs) using the fslmaths function within FSL. In addition, ten subcortical areas from the Harvard-Oxford probabilistic subcortical atlas (Desikan et al., 2006) were selected based on *a priori* hypotheses: caudate, amygdala, the hippocampus, nucleus accumbens, and putamen (left and right hemisphere regions were separate ROIs). Probabilistic subcortical masks were binarized using a 30% threshold, leaving ten subcortical ROIs that were added to the 400 cortical ones. Prior to analysis, all 410 ROIs were superimposed on the MNI152 template using FSLeyes (a function within FLS) to allow for visual inspection of the fit.

**Signal change extraction and missing data protocol**

The mean BOLD signal changes from the 410 ROIs were extracted for the three EVs across all subjects using the featquery tool (FSL). All extracted values were imported into SPSS v25 (IBM Corp, 2021). Backtracking of missing data sources was performed to assess outstanding issues with affected datasets. To account for missing values, the multiple imputation tool within SPSS was employed. Comparisons between pre- and post-multiple imputation means, ranges, and standard deviations were done to ensure no skewing of the data had taken place.

1. **Additional analyses examining group differences in DFH as a function of task (dampen vs. view negative)**

Follow up 2 (Group) x 2 (Task) ANOVAs revealed significant interactions for HC_Train_ vs BD and UR vs BD (F(1, 119) = 6.67, p=0.011, $\eta_{p}^{2}$ = .053) but not HC_Train_ vs UR or HC_Test_ (F(1, 107) & F(1, 63)= 0.18 & 0.00, p=0.67 & 0.99, $\eta_{p}^{2}$ = .002 & 0.000 respectively).

For t tests for individual DFHs (Emotion regulation and View Negative) for all groups, see Supplemental Table 3.

1. **Should the mode be trained on the patients with BD and/or the URs given the model trained on the HC_Test_ indicated poorer classification performance in these groups relative to HC_Train_? Alternatively should the model be trained** **on a mixture of data from patients with BD, URs and HCs? Could the model be considered biased against patients with BD as the model has not seen data from these individuals?**

We considered these important issues raised by a reviewer very important for discussion. We note though:

First, while the three (Group: HC_Test_, BD, UR) x two (Task: Emotion regulation, View negative) ANOVA on the DFH data revealed a significant Group-by-Task interaction, follow up 2 (Group) x 2 (Task) ANOVAs showed that this was because of significant interactions for HC_Test_ vs BD and UR vs BD. While it is true that the models have not seen the data of the patients with BD, they have also not seen the data of the HC_Test_ or URs either.

Second, the interaction for HC_Test_ vs UR was not significant. Similarly, HC_Test_ and UR did not significantly in classification accuracy while HC_Test_ and BD did. In short, patients with BD were showing more pronounced difficulty during dampening emotion than *both* the other participant groups who had also not been involved in the training of the model (and who did not differ).

Third, the goal of this project was to use SVM to identify a statistical model of dampening emotion vs. viewing emotion in the *healthy* brain and then examine the implications of that mode in independent samples. Including URs and particularly patients with BD in the training model would, by definition, not lead to the identification of a model of healthy brain function.

Fourth, we do not believe it accurate to consider these models “biased against bipolar disorder as they have never seen these individuals” because, as noted above, while it is true the models have not seen the data of the patients with BD they have also not seen the data of the HC_Test_ or URs either.

Having said this, we also developed an additional two SVMs, one trained on the URs and one trained on half of the patients with BD.

With respect to the SVM trained on the UR data: This did not reveal differences in accuracy with respect to HCs vs patients with BD. However, a 2 (group) x 2 (DFH: Dampen vs View) ANOVA did reveal a significant interaction (p = 0.032) – the patients with BD differentiated the conditions to a lesser extent than the HCs. It should be noted that both the HCs and the patients with BDs showed less differentiation than the UR training set. However, the degree of less differentiation was far more marked for the patients with BD (p<0.001; $\eta_{p}^{2}$ = .11) than the HCs (p=0.013; $\eta_{p}^{2}$ = .04).

With respect to the SVM trained on half of the sample of patients with BP: This revealed markedly poorer accuracy for dampen trials for the test patients with BP relative to HCs (59% vs 82%; p=0.009).

**Supplementary Table 1: Accuracy, sensitivity and specificity results for the participant groups.**

|  | HC_test_ | UR | BD | HC_train_ | χ2(df[2]) | p |
| --- | --- | --- | --- | --- | --- | --- |
| Overall Accuracy | 0.75 | 68.91 | 64.53 | 91.43 | 2.35 | 0.31 |
| Sensitivity (for ER) | 0.80 | 63.51 | 53.49 | 94.29 | 6.80 | 0.03 |
| Specificity for View Negative | 0.70 | 74.32 | 75.58 | 88.57 | 0.36 | 0.83 |

Note: The χ2 analyses were conducted on the data from the HC_test_, UR and patients with BD groups only**.**

**Supplementary Table 2: Table of features identified by the classifier**

| Hemisphere | Region | Schaefer Number | Greater response to |
| --- | --- | --- | --- |
| L | fronto polar | 176 | Emotion regulation |
| L | Inferior frontal cortex | 135, 136 | Emotion regulation |
| L | Temporoparietal juction | 163 | Emotion regulation |
| L | Superior Temporal | 72, 156, 160, 162 | Emotion regulation |
| L | Temporal pole | 119 | Emotion regulation |
| L | Medial temporal | 2 | View negative |
| R | Superor frontal | 118 | Emotion regulation |
| R | Intraparietal | 83 | Emotion regulation |
| R | Insula | 35 | Emotion regulation |
| R | Superior Temporal | 73 | Emotion regulation |
| R | Middle temporal | 84 | Emotion regulation |
| R | fusiform | 3 | Emotion regulation |
| R | Occipital | 16 | Emotion regulation |
| R | Parietal | 136 | View negative |

Note: Each Schaefer number corresponds to a region (feature); i.e., Superior temporal cortex involved 4 features.

**Supplementary Table 3: t tests for individual DFHs (Emotion regulation and View Negative) for all groups.**

|  |  | Patients with BD | | |  | URs | | |  | HC_Test_ | | |
| --- | --- | --- | --- | --- | --- | --- | --- | --- | --- | --- | --- | --- |
|  |  | t | df | p |  | t | df | p |  | t | df | p |
| URs | Emotion Regulation | -1.75 | 158 | 0.08 |  |  |  |  |  |  |  |  |
|  | View Negative | 0.59 | 158 | 0.55 |  |  |  |  |  |  |  |  |
| HC_Test_ | Emotion Regulation | -2.86 | 114 | 0.00 |  | -0.95 | 102 | 0.34 |  |  |  |  |
|  | View Negative | -1.17 | 114 | 0.25 |  | -1.45 | 102 | 0.15 |  |  |  |  |
| HC_Train_ | Emotion Regulation | -2.25 | 119 | 0.03 |  | -0.28 | 107 | 0.78 |  | 1.15 | 63 | 0.13 |
|  | View Negative | -0.18 | 119 | 0.86 |  | -0.66 | 107 | 0.51 |  | 1.18 | 63 | 0.12 |

**Supplementary Table 4: Significant Group x Task interactions for different selections of patients with BD as a function of their prescribed medications.**

| Inclusion/exclusion criterion | N (BD) | df | F | Sig. | 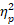   \|  \| \| --- \| |
| --- | --- | --- | --- | --- | --- | --- |
| Excluding those on any medication | 19 | 2, 120 | 3.44 | 0.035 | 0.054 |
| Including only those on medication | 66 | 2, 167 | 5.49 | 0.005 | 0.062 |
| Excluding those on anti-depressant medication | 66 | 2, 167 | 4.97 | 0.008 | 0.056 |
| Excluding those on anti-psychotic medication | 68 | 2, 169 | 7.83 | 0.001 | 0.085 |
| Excluding those on ant-convulsant medication | 54 | 2, 155 | 5.86 | 0.004 | 0.070 |
| Excluding those on lithium | 45 | 2, 146 | 4.88 | 0.009 | 0.063 |

Key to Supplemental Table 4: N(BD) = Number of patients with BD according to the exclusion/inclusion criterion

**Supplementary Tables 5: Changes in functioning over the 16-month follow-up time in patients with BD.**

| **Table 5.1.** Change in functioning over the 16-month follow-up time in patients with BD | |  |  |
| --- | --- | --- | --- |
|  |  | Wilcoxon Signed Ranks | |
|  | Median [IQR] | Z | *p* |
| FAST total score, baseline | 11 [4-21.5] | -2.90 | 0.004 |
| FAST total score, follow-up | 6 [4-13] |  |  |

| **Table 5.2.** Mood episodes during the 16-month follow-up time |  |
| --- | --- |
| BD-, n (%) | 19 (32%) |
| BD+, n (%) | 41 (68%) |
| Mood episodes between baseline and follow-up in BD+ patients | Median [IQR] |
| Total number of episodes | 2 [1-3.5] |
| Total duration in episode | 117 [55-197] |
| Depressive episodes, no. | 1 [1-2] |
| Depressive episodes, days | 85 [30-162] |
| (Hypo)manic episodes, no. | 0 [0-1] |
| (Hypo)manic episodes, days | 0 [0-17] |
| Abbreviations: BD- = patients who remained in remission during the follow-up time; BD+ = patients who experienced at least one mood episode during the follow-up time; IQR = interquartile range | |

**Tables 5.3: Regression results for change in function level (FAST scores)**

| **Model Summary** | | | | | | | | |  |  |  |
| --- | --- | --- | --- | --- | --- | --- | --- | --- | --- | --- | --- |
| Model | | R | R Square | | Adjusted R Square | | Std. Error of the Estimate | |  |  |  |
| 1 | | .324^a^ | .105 | | .086 | | 10.18 | |  |  |  |
| a. Predictors: (Constant), emotion regulation distance from ) hyperplane (DFH_ER_) | | | | | | | | |  |  |  |
| **ANOVA^a^** | | | | | | | | | | | |
| Model | | | | Sum of Squares | | df | | Mean Square | | F | Sig. |
| 1 | Regression | | | 581.31 | | 1 | | 581.31 | | 5.61 | .022^b^ |
|  | Residual | | | 4972.61 | | 48 | | 103.60 | |  |  |
|  | Total | | | 5553.92 | | 49 | |  | |  |  |
| a. Dependent Variable: FAST total change | | | | | | | | | | | |
| b. Predictors: (Constant), DFH_ER_ | | | | | | | | | | | |

| **Coefficients^a^** | | | | | | | | | | | |  |
| --- | --- | --- | --- | --- | --- | --- | --- | --- | --- | --- | --- | --- |
| Model | | Unstandardized Coefficients | | | | Standardized Coefficients | | t | | Sig. | |  |
|  |  | B | | Std. Error | | Beta | |  |  |  |  |  |
| 1 | (Constant) | -3.21 | | 1.47 | |  | | -2.18 | | .034 | |  |
|  | DFH_ER_ | -1.66 | | .70 | | -.32 | | -2.37 | | .022 | |  |
| a. Dependent Variable: FAST total change | | | | | | | | | | | |  |
| **Excluded Variables^a^** | | | | | | | | | | | | |
| Model | | | Beta In | | t | | Sig. | | Partial Correlation | | Collinearity Statistics | |
|  |  |  |  |  |  |  |  |  |  |  | Tolerance | |
| 1 | HDRS | | -.18^b^ | | -1.33 | | .19 | | -.19 | | .997 | |
|  | YMRS | | -.05^b^ | | -.37 | | .71 | | -.05 | | .991 | |
|  | Sex | | .05^b^ | | .34 | | .74 | | .05 | | .931 | |
|  | Bipolar type | | .03^b^ | | .24 | | .81 | | .04 | | .997 | |
|  | Age | | -.001^b^ | | -.006 | | .995 | | -.001 | | .945 | |
|  | DFH_VN_ | | .18^b^ | | .78 | | .44 | | .11 | | .34 | |
|  | Days between MR & episode data | | -.20^b^ | | -1.37 | | .18 | | -.20 | | .90 | |
| a. Dependent Variable: FAST total change | | | | | | | | | | | | |
| b. Predictors in the Model: (Constant), DFH_ER_ | | | | | | | | | | | | |

Key: HDRS: Hamilton Depression Rating Scale; YMRS: Young Mania Rating Scale; DFH_VN_: Distance from hyperplane for view negative trials.

***Total number of relapse episodes***

| **Model Summary** | | | | |
| --- | --- | --- | --- | --- |
| Model | R | R Square | Adjusted R Square | Std. Error of the Estimate |
| 1 | .42^a^ | .18 | .16 | 1.89 |
| a. Predictors: (Constant), Days between MR and episode data | | | | |

| **ANOVA^a^** | | | | | | |
| --- | --- | --- | --- | --- | --- | --- |
| Model | | Sum of Squares | df | Mean Square | F | Sig. |
| 1 | Regression | 42.18 | 1 | 42.18 | 11.78 | .001^b^ |
|  | Residual | 196.87 | 55 | 3.58 |  |  |
|  | Total | 239.05 | 56 |  |  |  |
| a. Dependent Variable: Total number of episodes since baseline MR | | | | | | |
| b. Predictors: (Constant), Days between MR and episode data | | | | | | |

| **Coefficients^a^** | | | | | | |
| --- | --- | --- | --- | --- | --- | --- |
| Model | | Unstandardized Coefficients | | Standardized Coefficients | t | Sig. |
|  |  | B | Std. Error | Beta |  |  |
| 1 | (Constant) | -.83 | .79 |  | -1.05 | .298 |
|  | Days between MR and episode data | .01 | .002 | .42 | 3.43 | .001 |
| a. Dependent Variable: Total number of episodes since baseline MR | | | | | | |

| **Excluded Variables^a^** | | | | | | |
| --- | --- | --- | --- | --- | --- | --- |
| Model | | Beta In | t | Sig. | Partial Correlation | Collinearity Statistics |
|  |  |  |  |  |  | Tolerance |
| 1 | HDRS | .18^b^ | 1.45 | .15 | .19 | .998 |
|  | YMRS | .21^b^ | 1.78 | .08 | .24 | .995 |
|  | Sex | .20^b^ | 1.67 | .10 | .22 | .999 |
|  | Bipolar type | .05^b^ | .39 | .70 | .05 | .998 |
|  | Age | .19^b^ | 1.57 | .12 | .21 | .981 |
|  | DFH_ER_ | -.20^b^ | -1.57 | .12 | -.21 | .937 |
|  | DFH_VN_ | -.16^b^ | -1.32 | .19 | -.18 | .993 |
| a. Dependent Variable: FU_Total_no_ep_sinceBL | | | | | | |
| b. Predictors in the Model: (Constant), Days_between_MR_episodedata | | | | | | |

Key: HDRS: Hamilton Depression Rating Scale; YMRS: Young Mania Rating Scale; DFH_VN_: Distance from hyperplane for view negative trials.

Supplemental Figure 1: The hyperplane that maximizes the distances from the support vectors corresponding to individual participants’ responses during emotion regulation (“Dampen”) and viewing negative images. Note only two features are shown in the Figure (X_1_ and X_2_). However, the SVM conducted on the fMRI data here is multidimensional (and led to a hyperplane involving 18 features). Red two-headed arrow: Distance from hyperplane (DFH). Dots: Each dot corresponds to an individual participant’s response during emotion regulation (red/orange dots) and when viewing negative images (green dots) as a function of the schematic two features X_1_ and X_2_.


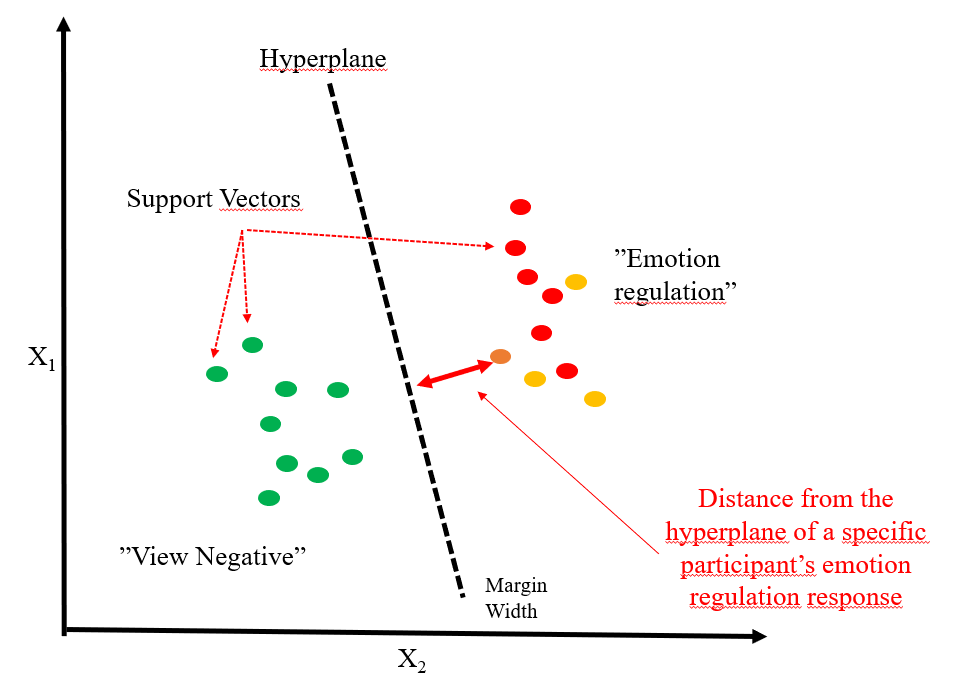


**Supplemental References**

Banks, S. J., Eddy, K. T., Angstadt, M., Nathan, P. J. & Phan, K. L. (2007). Amygdala-frontal connectivity during emotion regulation. *Social Cognitive Affective Neuroscience,* *2,* 303-12. doi:10.1093/scan/nsm029.

Buhle, J. T., Silvers, J. A., Wager, T. D., Lopez, R., Onyemekwu, C., Kober, H., . . . Ochsner, K. N. (2014). Cognitive reappraisal of emotion: a meta-analysis of human neuroimaging studies. *Cerebral Cortex,* *24,* 2981-90. doi:10.1093/cercor/bht154.<http://cercor.oxfordjournals.org/content/24/11/2981.full.pdf>

Corp, I. (2021). IBM SPSS Statistics for Windows, Version 25.0. . IBM Corp: Armonk, NY.

Desikan, R. S., Ségonne, F., Fischl, B., Quinn, B. T., Dickerson, B. C., Blacker, D., . . . Killiany, R. J. (2006). An automated labeling system for subdividing the human cerebral cortex on MRI scans into gyral based regions of interest. *Neuroimage,* *31,* 968-80. doi:10.1016/j.neuroimage.2006.01.021.

Hamilton, M. (1967). Development of a rating scale for primary depressive illness. *Br J Soc Clin Psychol,* *6,* 278-96. doi:10.1111/j.2044-8260.1967.tb00530.x.

Jenkinson, M., Beckmann, C. F., Behrens, T. E., Woolrich, M. W. & Smith, S. M. (2012). FSL. *Neuroimage,* *62,* 782-90. doi:10.1016/j.neuroimage.2011.09.015.

Kjærstad, H. L., Eikeseth, F. F., Vinberg, M., Kessing, L. V. & Miskowiak, K. (2021). Neurocognitive heterogeneity in patients with bipolar disorder and their unaffected relatives: associations with emotional cognition. *Psychological Medicine,* *51,* 668-679. doi:10.1017/s0033291719003738.

Kjærstad, H. L., Macoveanu, J., Knudsen, G. M., Frangou, S., Phan, K. L., Vinberg, M., . . . Miskowiak, K. W. (2023). Neural responses during down-regulation of negative emotion in patients with recently diagnosed bipolar disorder and their unaffected relatives. *Psychological Medicine,* *53,* 1254-1265. doi:10.1017/s0033291721002737.

Kjærstad, H. L., Poulsen, E., Vinberg, M., Kessing, L. V. & Miskowiak, K. W. (2022). Differential trajectory of cognitive functions in neurocognitive subgroups of newly diagnosed patients with bipolar disorder and their unaffected first-degree relatives - A longitudinal study. *Journal of Affective Disorders,* *311,* 115-125. doi:10.1016/j.jad.2022.05.045.

Lang, P. J., Bradley, M. M. & Cuthbert, B. N. (1997). *International affective picture system (IAPS): Technical manual and affective ratings*. NIMH Center for the Study of Emotion and Attention.

Rosa, A. R., Sánchez-Moreno, J., Martínez-Aran, A., Salamero, M., Torrent, C., Reinares, M., . . . Vieta, E. (2007). Validity and reliability of the Functioning Assessment Short Test (FAST) in bipolar disorder. *Clinical Practice and Epidemiology in Mental Health,* *3,* 5. doi:10.1186/1745-0179-3-5.

Schaefer, A., Kong, R., Gordon, E. M., Laumann, T. O., Zuo, X. N., Holmes, A. J., . . . Yeo, B. T. T. (2018). Local-Global Parcellation of the Human Cerebral Cortex from Intrinsic Functional Connectivity MRI. *Cerebral Cortex,* *28,* 3095-3114. doi:10.1093/cercor/bhx179.

Wing, J. K., Babor, T., Brugha, T., Burke, J., Cooper, J. E., Giel, R., . . . Sartorius, N. (1990). SCAN: Schedules for Clinical Assessment in Neuropsychiatry. *Archives of General Psychiatry,* *47,* 589–593. doi:<https://doi.org/10.1001/archpsyc.1990.01810180089012>.

Woolrich, M. W., Ripley, B. D., Brady, M. & Smith, S. M. (2001). Temporal autocorrelation in univariate linear modeling of FMRI data. *Neuroimage,* *14,* 1370-86. doi:10.1006/nimg.2001.0931.

Young, R. C., Biggs, J. T., Ziegler, V. E. & Meyer, D. A. (1978). A rating scale for mania: reliability, validity and sensitivity. *Br J Psychiatry,* *133,* 429-35. doi:10.1192/bjp.133.5.429.
